# Evolution of the Concepts of Architecture and Supramolecular Dynamics of the Plasma Membrane

**DOI:** 10.3390/membranes13060547

**Published:** 2023-05-24

**Authors:** Carolina Campos Muñiz, Francisco José Fernández Perrino

**Affiliations:** 1 Department of Health Sciences, Universidad Autónoma Metropolitana Iztapalapa, Av. San Rafael Atlixco 186, Col. Vicentina, Iztapalapa, Mexico City 09340, Mexico; 2 Department of Biotechnology, Universidad Autónoma Metropolitana-Iztapalapa, Av. San Rafael Atlixco 186, Col. Vicentina, Iztapalapa, Mexico City 09340, Mexico

**Keywords:** membrane organization models, lipid bilayer, dynamic membranes, plasma membrane organization, structure of membranes

## Abstract

The plasma membrane (PM) has undergone important conceptual changes during the history of scientific research, although it is undoubtedly a cellular organelle that constitutes the first defining characteristic of cellular life. Throughout history, the contributions of countless scientists have been published, each one of them with an enriching contribution to the knowledge of the structure-location and function of each structural component of this organelle, as well as the interaction between these and other structures. The first published contributions on the plasmatic membrane were the transport through it followed by the description of the structure: lipid bilayer, associated proteins, carbohydrates bound to both macromolecules, association with the cytoskeleton and dynamics of these components.. The data obtained experimentally from each researcher were represented in graphic configurations, as a language that facilitates the understanding of cellular structures and processes. This paper presents a review of some of the concepts and models proposed about the plasma membrane, emphasizing the components, the structure, the interaction between them and the dynamics. The work is illustrated with resignified 3D diagrams to visualize the changes that occurred during the history of the study of this organelle. Schemes were redrawn in 3D from the original articles...

## 1. Introduction

The plasma membrane (PM) is a perimeter organelle that acts as a selective barrier, separating cellular constituents from their environment; it establishes the first defining characteristic of cellular life. It is made up of three main components, lipids, proteins, and carbohydrates, in a variable mass ratio that depends on the cell type. Sugars are incorporated into the membrane as post-translational modifications of proteins and lipids (in the form of glycoproteins and glycolipids, respectively). The architecture of the membranes, the physical and chemical properties of the macromolecules, and the interactions between them determine functions such as selective transport, cell recognition, signaling (signal transduction), and the compartmentalization of cellular processes. The PM has been extensively studied to understand its architecture, the function of each component, and its interaction with other structures, such as membranes, cytoskeleton, and extracellular matrix. Our knowledge of PM has undergone important conceptual changes throughout the history of scientific research [1,2,3,4].

With technological progress, it has been possible to a great extent to understand the structure, location, interactions with other structures and function of the PM. However, to date, there is no apparatus or equipment in which the ultrastructure of the PM can be observed [2]. The enriching biophysical and biochemical advances in the structure have been translated into “graphic configurations” such as schemes, diagrams, images, graphs, etc., published in scientific research articles. In this way, knowledge is made accessible, facilitating the understanding of the new concepts [5].

There are membranes, such as those of plants and fungi, with special characteristics. The present review describes rather general models, without going into particular aspects. The purpose of this review is to describe some models of the PM throughout history (as well as some concepts related to it), with the idea of showing how knowledge of the PM has evolved. It begins with the identification of its components, followed by its function and interactions with various macromolecules (already involving the participation of the PM in the translation of cellular signals). The contribution of biophysicists, physicists, chemists, biologists, and people not involved in science (such as Pockels) is highlighted. At the same time, the previously published diagrams were redesigned in 3D versions, with the idea of comparing the models throughout the history of the PM study.

## 2. Evolution of the Concept of Plasma Membrane or Biomembrane

The origin of studies of the cell membrane is not well defined; however, it is assumed that it began with the studies of Hewson [6], who in 1773 analyzed erythrocytes in water and observed that the shape of the cell under the microscope changed from discoid to spherical. With too much water, the erythrocytes simply dissolved, a process now known as hemolysis. Hewson was the first to show the osmotic swelling and shrinkage of erythrocytes, thus deducing the existence of a cell membrane as a structure that surrounds a liquid or protoplasm. These contributions were ignored; however, Hewson was called the “father of the hematology” [2].

With these results it was evident that the cell was isolated from the environment that surrounded it, thus beginning the history of the study of the membranes, that followed two different paths: firstly research with lipid monolayers (oil in water), and secondly with living cells. The first experiment of oil on water is attributed to Franklin in 1772 [7], which was refined and perfected by Pockels, an extraordinary woman who designed an apparatus with homemade utensils to measure surface tension and fat layers. She describes her first experiments in a letter she sends to Rayleigh in 1891 (and he, in turn, sends it to the journal Nature) [8]. Beginning in 1894, Pockels published the results of her experiments on surface tension by using monolayers of olive oil [9]. The device designed by Pockels was taken up and used by Langmuir in 1917 [10].

## 3. Concept of the Plasmatic Membrane and Transport

**Figure 1:**
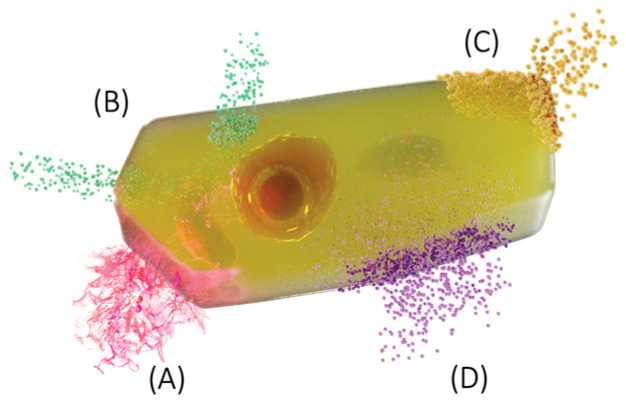
Transport across the membrane. First reports of the existence of a semi-permeable cell membrane. (**A**) Permeability of pigments in protoplasts [11]. (**B**) Artificial membranes, osmotic pressure, semi-permeable membranes [12]. (**C**) Permeability of small molecules in protoplasts [14]. (**D**) Transport of molecules depends on the solubility of fats [17].

The origin of the concept of the PM is ambiguously identified; however, it is attributed to Nägeli and Cramer [11] (Figure 1A), who had been working with algae, fungi, mosses, and plants. On the one hand, they observed that the cell surface was impermeable to the pigments added to the solution that surrounded it. From the experiments they carried out on osmosis, they concluded that the PM gives the cell osmotic properties, which is why they called it the superficial layer or invisible film (“cell membrane”). On the other hand, Plower in 1931 gave it the term “plasmalemma” [2].

Pfeffer [12] used an apparatus designed by himself, called Pfeffer Zelle or Pfeffer Cell, which used an artificial membrane of copper ferrocyanide as a model of a plant membrane to measure the amount of pressure inside a plant cell. He defined osmotic pressure as the point at which equal amounts of fluid enter and leave the membrane and that the components and the size of the membrane pores play an important role in the osmosis of plant cells, which stops the passage of substances. So, he deduced that the cell barrier should be thin and semi-permeable [13] (Figure 1B).

In 1884, De Vries [14], in microscopic studies where plant cell protoplasts were observed, detected that they were permeable to water, but not to larger macromolecules such as sucrose, which suggested the presence of a biological membrane or an invisible sheet that delimits the cell. These insights marked the beginning of the study of PM (Figure 1C).

One of the first indications that the membranes were composed of fats was reported in 1888 by Quincke [15], who observed the spherical shape of a cell immersed in water, which, when broken, formed two smaller spheres. He compared this behavior with that of petroleum and intuited that the nature of PM was fatty and that, akin to petroleum in water, it must be a thin film of oil that behaves like a semi-permeable membrane approximately 100 nm thick.

Another approach to add to knowledge of the structure of the PM was proposed by Overton [16]. Overton’s main contribution to the understanding of membranes involved permeability studies; where he described that, in addition to water, other molecules are able to cross membranes; the fastest to pass being small molecules, and that, the entry of any molecule into the cell is governed by its solubility in fats. He observed that most of the molecules that crossed the membrane did so passively, following the concentration gradient. However, not all the molecules that crossed had this behavior, because some passed against the concentration gradient; we now know this as active transport (Figure 1D).

## 4. Membrane Models and Contributions to the Concept of the Membrane

### 4.1. Lipid Structure

#### 4.1.1. Lipid Monolayer

**Figure 2:**
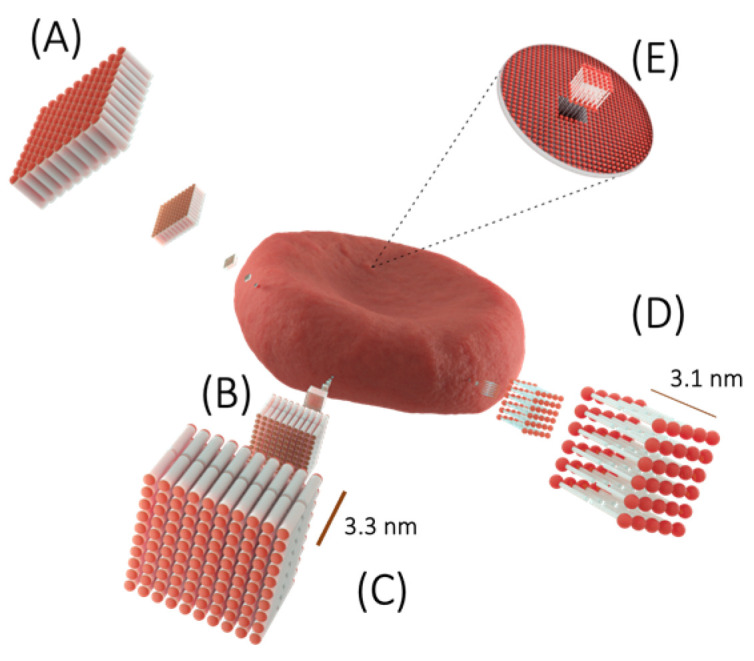
Lipid structure of the plasma membrane. (**A**) Langmuir proposes a lipidic monomolecular membrane [10]. (**B**) The membrane is composed of a lipid bilayer [18]. (**C**) Fricke (Fricke, 1923, 1925) estimates the thickness of the erythrocyte membrane, although it does not consider the hydrophilic group of membrane lipids and the calculated value was from its hydrophobic nucleus (3.3 nm). (**D**) Grendel calculated the membrane thickness to be 3.1 nm [26]. (**E**) Representation of a fragment of the plasma membrane.

From the studies of Quincke [15] and Overton [16,17], it was already known that cell membranes were composed of fats. Langmuir in 1917 [10] worked with fats in a solution of benzene and formalin in which benzene-fat monolayers are formed. This experiment was carried out in a water–air interface, and he observed that when the benzene evaporates the molecules remain as a layer of fats of one molecule wide. From these observations, the existence of a monomolecular oil membrane was proposed (Figure 2A). The samples were measured with the apparatus implemented by the German scientist Agnes Pockels (1891), who later perfected Langmuir bearing the name “Langmuir–Blodgett trough” which is still in use to this day. In 1889, Rayleigh examined the distribution of oil on the surface of the water, measuring its thickness using the same apparatus modified by Langmuir.

#### 4.1.2. Lipid Bilayer

The monomolecular membrane of oil reported by Langmuir in 1917 and the measurements of the thickness of fats (term used in the articles of those years) obtained by Lord Rayleigh in 1889 were the basis that allowed Gorter and Grendel in 1925 [18] to determine the approximate area and thickness of a cell membrane. One success was the choice of cell model: erythrocytes, since they lack organelles. The “ghosts” of a known number of these cells were isolated and the biconcave shape of the erythrocytes was measured using the Knoll formula [19]. Once the membranes were obtained, they used the Langmuir–Blodgett trough apparatus to expand the membranes on an aqueous surface. They found that the surface area of the fats was approximately twice the area of the erythrocyte membranes. The authors correctly inferred that the erythrocyte membrane is made up of a fat bilayer, with a thickness of 5 to 6 nm (Figure 2B). This experiment was key to understanding the structure of biological membranes. Today, it is known that Gorter and Grendel underestimated one-third of the surface area of erythrocytes and one-third of the amount of lipids present in PM. In addition, they did not consider the significant proportion occupied by erythrocyte membrane proteins. Fortunately, these errors cancelled each other out, so the authors’ conclusion was correct, even though their data were not.

#### 4.1.3. Cell Bilayer Thickness

Knowing the membrane was a bilayer, Fricke [20,21] measured the electrical capacitance of the membranes of erythrocytes and various cell types to estimate the thickness of the membranes, arguing that the membranes were composed of molecules that he called “lipins” (such as lecithin, cholesterol, etc.) since the composition of the membrane was not known at that time. The result was approximately 0.81 μF/cm^2^, and the dielectric constant was 3.3 × 10^−7^ cm. He calculated that the capacitance corresponds to a thickness of 3.3 nm, which is roughly equivalent to the length of a fat molecule. However, Fricke did not consider the fact that lipids have a sizable hydrophilic head group, and, therefore, in his experimental plot, he did not measure the total thickness of the erythrocyte membrane, only that of its hydrophobic nucleus (Figure 2C).

The concept of lipid was not always used: in 1926, Sperry used it for the first time [22]. Before this, the most used terms were fats or lipoids. The term lipid is definitively adopted in 1960. During that time some lipid structures were determined, including phosphatidylcholine in 1874 by the French scientist Gobley [23]. On the other hand, the chemical structure of cholesterol was reported in 1927 by Windaus and Wieland (both Nobel Prize winners) [24], while in 1937 it was reported by Crowfoot (also a Nobel Prize winner) and Bernal through X-ray diffraction of the crystal structure of the compound [25]. The sense of diversity already existed in this period due to the exponential rate of discovery of different fatty acids that make up the acyl chains of lipids.

Later, Grendel [26] analyzed the total of extracted “lipoids”, in which he made measurements of the surfaces occupied by the individual constituents and calculated that the bilayer would have an average thickness of 3.1 nm (Figure 2D). Later still, Danielli and Davson in 1935 [27] examined the surface potentials in lipid films, and the estimated thickness result was 12 nm. For their part, Cole and Curtis in 1936 reported the measurement of electrical impedance in the membrane, which would be equivalent to a thickness of 5 nm and showed that the capacitance of the membrane is remarkably uniform from one cell type to another [28].

### 4.2. Lipid Bilayer and Membrane Proteins

#### 4.2.1. The Lipid Bilayer Contains Proteins

**Figure 3:**
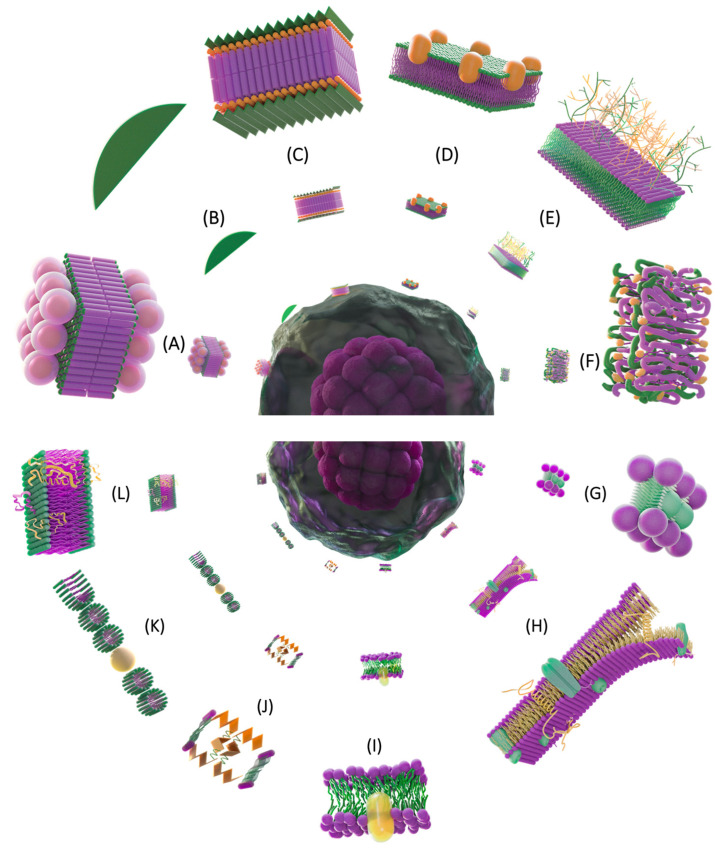
Lipid–protein structure of the plasma membrane. (**A**) Danielli and Davson [27], propose the “sandwich” model with a thickness of 10 nm considering Langmuir’s lipid bilayer, covered on both sides by “globules”. (**B**,**C**) Robertson (Robertson, 1957), using electron microscopy, proposes the unitary membrane model with a thickness of between 7.5 and 10 nm. Like Danielli and Davson (1935) he proposes proteins on the internal and external surfaces of the bilayer, but of the alpha helix and random coil type. (**D**) Mühlethaler, Moor and Szarkowski [40] observed proteins embedded in the outer lipid monolayer. (**E**) Bennett [41], and Fawcett [42] show the glycocalyx in intestinal cells. (**F**) Benson [43] publishes the protein subunit model, consisting of lipoprotein subunits of interlinked polypeptide and fatty acyl chains. The ionic groups of lipids and proteins are confined to the surfaces of water. (**G**) Vanderkooi and Green [47] propose the protein crystal model protein for membranes, which shows a double layer of proteins (spheres) with lipid bilayer regions. (**H**) Branton [49,50], by using the fracture of frozen membrane technique, observed bulges and depressions that corresponded to proteins, found immersed in lipids. (**I**) Chapman and Penkett [51] propose the concept of fluidity, as the movement of the hydrocarbon chains of fatty acids. (**J**) Lenard and Singer [53] propose the mosaic model of lipids and proteins. The ionic heads of the lipids are shown next to the ionic chains of the protein on the surface of the membrane, and the structural protein’s nonpolar side chains are found in the interior. (**K**) Lucy [55] proposes a micellar model in which proteins are inserted into the micelles. (**L**) Singer [54] proposes the protein mosaic model where the globular proteins in yellow (polypeptide chain) are amphipathic molecules and highly polar groups exposed on the outer surfaces of the membranes.

Soon, after Gorter and Grendel proposed the bilayer model in 1925 [18], it became clear that an important feature of membrane structure was the lipid bilayer; however, this fact could not explain all the properties of PM, especially those related to surface tension, solutes permeability, and electrical resistance. To explain these differences, Danielli and Harvey [29] performed indirect experiments on membrane properties such as surface tension and thickness calculations, evidencing the need for an additional factor to explain the attenuation of this parameter in biological membranes. The authors appropriately attributed it to the presence of protein. In the same year, Danielli and Davson proposed that biological membranes consisted of a triple layer; a lipid bilayer, covered on both sides by adsorbed globular proteins; most of them soluble in water, so the authors proposed the classic “sandwich” model [27] (Figure 3A).

Years later, Schmitt and Palmier [30], measuring the thickness using X-ray diffraction, found periodicities of 6.3 nm and 160 nm in dry motor nerve roots. In later studies, the same authors, measuring the length of an extended phospholipid molecule, found a thickness of about 3.5 nm, which would be the total for the model of Danielli and Davson [27]; the protein-lipid ensemble and the thickness of the membrane should be of the order of 10 nm.

#### 4.2.2. Composition and Types of Lipids in the Plasmatic Membrane

Although it was already known that the membranes were composed of a lipid bilayer, it was not known whether they were formed by a single type or different lipids, so various membranes were analyzed to determine the types of lipids that constituted them. In 1929, Grendel [26] analyzed the total extracted lipids in erythrocytes, from measurements of the surfaces occupied by the individual constituents and concluded that the relative contributions (in terms of extended area) were as follows: cholesterol 36%, cephalin-lecithin 50%, and sphingomyelin 13%.

Other phospholipids were later characterized, such as cardiolipin [31], phosphatidylethanolamine (PE), phosphatidylserine (PS) in 1942 [32], phosphatidylglycerol (PG) [33], and, finally, phosphatidylinositol (PI) [34]. Ceramide and ganglioside lipid structures were described in the 1960s [35,36,37].

On the other hand, de Gier and Van Deenen [38] compared the lipid composition of erythrocyte ghosts (only of a cell membrane) from six mammalian species and observed little variation in total lipids and cholesterol. However, large differences were present in the percentages of phosphatidylcholine, phosphatidylethanolamine, and phosphatidylserine. Until then, the distribution of phospholipids in bilayers was unknown.

#### 4.2.3. Membrane Unit or Unitary Model

With the introduction of the electron microscope, a new era in PM studies began. Robertson [39], makes ultrathin cuts in cells in cross-section after fixation with osmium tetroxide and recognizes in electron micrographs the membrane as a similar fine structure in plant, animal, and protozoan cells. On a quantitative basis provided from the ultrastructural data, he observed two dark lines separated by a faintly stained central zone in which each dark section was 2 nm thick and the clear layer 3.5 nm, for a total of 7.5 to 10 nm (Figure 3B) (similar data reported by Schmitt and Palmer in 1940 [30]). He described the membranes as having a characteristic “trilaminar” appearance, which he observed in different cells, suggesting that all cell membranes shared a common structure, which he called a “unit membrane”. According to this model, the outer two darker lines were the protein layers and the inner region the lipid bilayer [39]. When this model was proposed, which seemed to agree perfectly with Danielli and Davson’s 1935 model, Robertson suggested that the lightly stained space (between the two dark lines of the trilaminar pattern) contained the hydrophobic region of the lipid molecules, which were not easily stained, and the dark region was constituted by proteins, proposing the trilaminar model (Figure 3C) [39].

#### 4.2.4. Peripheral Proteins

In studies on thylakoid membranes, Mühlethaler, Moor, and Szarowski [40], using the Freeze etching technique, assumed the presence of particles that they called multienzyme complexes, which they observed on the surface of the lipid bilayer and to partially penetrate between the lipids. These proteins are embedded in the outer layer of the thylakoids. (Figure 3D).

#### 4.2.5. Cell Surface

In addition to the lipid bilayer and proteins described in the previous models, a structure called glycocalyx was observed by Bennett in 1963, who suggested that each cell could have a carbohydrate coating on the external surface of the PM [41]; this hypothesis was confirmed in many cell types. This filamentous network was called “the sugar layer”, and this matrix was studied under the electron microscope by Fawcett [42]. This author succeeded in photographing the intestinal microvilli, observing the structural arrangement, branched in the form of a network, whose filaments were observed from 2.5–5 nm thick and 0.1 to 0.5 μm length, arranged radially. In turn, Ito in 1974 observed the glycocalyx in the periphery of the intestine as a uniform layer of filamentous material, less than 0.1 μm, and sometimes greater than 0.5 μm in thickness (Figure 3E).

#### 4.2.6. Models of the 1960s: Membrane Proteins

Until this point, the concept of the membrane still did not contain elements that would allow an understanding of the molecular structure, shape, interaction of proteins with lipids, and some functions. During the 1960s, several contemporary models were proposed, as well as the concept of PM fluidity. The study of membranes was in its early stages. It should not be surprising that even the existence of the lipid bilayer was doubted by some researchers, since two models replaced it: the one for photosynthetic thylakoids (using lipoprotein subunits) proposed by Benson [43], and the other for mammalian mitochondria proposed by Green [44,45]. These models received special attention due to the recognition of the researchers who proposed them. Benson was a world-renowned plant physiologist, as his early work was the discovery of the Calvin Benson cycle (photosynthetic dark reactions) and Green, who was a leader in the study of mitochondria, possessed an excellent reputation, based on the study of mitochondrial electron transport and oxidative phosphorylation. Therefore, both Benson and Green were experts in highly specific and sophisticated membranes. The membranes of these organelles are not “typical”; they are in the upper range of protein–lipid ratios relative to PM [44,45]. The models of both authors are described below.

##### Protein Subunit Model

Benson published a remarkable membrane model, based on his studies of the thylakoid membranes [43]. He proposed that protein chains intercalate and wrap around the fatty acid chains of membrane phospholipids, forming discrete lipoprotein subunits that pack into each other in the plane of the membrane. In this model, the lipids are not in classical bilayer form because they are interrupted by associated protein chains. The polar head groups of lipids and ionic residues of the proteins are in contact with water on both sides of the membrane, whereas the hydrophobic residues of the protein are located on the inside of the membrane, away from water contact (Figure 3F).

##### The Lipoprotein-Repeating Unit’s Model and the Protein-Crystal Model

In the same year, Green and Perdue [46] proposed that the membrane is a two-dimensional continuum system, the continuum being made up of nesting lipoprotein-repeating units. In this model, they explain the hydrophobic interactions between transmembrane proteins and lipids; however, they underestimate the presence and relevance of the lipid bilayer and proteins. Like Benson, they propose a similar model of lipoproteins based on their studies. On the other hand, Vanderkooi and Green [47] propose “the protein model crystal model for membranes” (Figure 3G), which consists of two layers of loosely packed proteins with clefts and interstices between the protein molecules being filled with non-polarity tails of lipid molecules, such that the polar heads of the lipids meet at the two water-membrane interfaces. Proteins are assumed to have more or less extensive hydrophobic regions, allowing hydrophobic bonds with lipid molecules. Protein molecules have limited contact regions with each other, which means that protein–protein interactions are involved in providing structural stability to the membrane. The lipid molecules do not make the membrane thinner than twice the protein layer by itself, since the nonpolar tails are within the boundaries of the protein layers. The authors mention that the presence of these lipid tails in the interstices can greatly enhance the mechanical strength of the membrane, which can affect its properties [47].

##### Fracture of Frozen Membranes

Subsequent to the cryofracture technique developed in the 1950s [48], Branton [49] performed a microscopy analysis on natural lipids and artificial membranes, in which he observed that the freezing of the membranes produced by the technique maintained the hydrophilic interactions on the surface, but cancelled the hydrophobic forces inside the membrane, allowing it to break between the two lipid layers. As the interior of the membrane became visible, some bulges were observed and on the opposite side of the monolayer where proteins were present, depressions were visualized. The bulges were called “globules”, which would soon be assimilated as membrane proteins (Figure 3H). Branton’s work in 1966 and 1971 [49,50] confirmed the existence of a lipid bilayer, as suggested by Gorter and Grendel [18], and the presence of proteins embedded in the lipid bilayer.

##### Concept of Fluidity

In addition to structural studies, the concept of fluidity was introduced in 1966 by Chapman and Penkett [51], for biomembranes as the “particular distribution of fatty acids for the diffusion and metabolic processes required by the cell”. For this important contribution, spectroscopy studies were performed detailing the molecular nature, mostly in the thermotropic transition phase of the long amphipathic chains. In other studies, they used pure phospholipids with infrared spectroscopy and nuclear magnetic resonance techniques; with them, they pointed out the molecular mobility associated to phospholipids. The data obtained by these authors showed that the hydrocarbon chains are flexible in the methyl groups, which showed a marked increase in the rotation of the isomers. The oscillations and rotational disorder of the methyl groups were markedly fluid at the end of the lipid chains; the fluidity of the membranes modulated the molecules found in the lipid bilayer, such as cholesterol (Figure 3I) [51,52].

##### Mosaic Model

With the knowledge of integral membrane proteins and the experimental data obtained by Branton [49], Lenard and Singer in the same year examined the conformation of proteins in cell membranes using the techniques of optical rotatory dispersion and circular dichroism [53]. These two techniques are invaluable in interpretation of the conformation of soluble proteins and polypeptides. Both authors carried out their experiments on erythrocyte and *Bacillus subtilis* membranes, concluding that between one-fourth and one-third of membrane proteins have a helical conformation. These researchers incorporated this finding into a new model for the cell membrane, based on two characteristics: the first is the presence of alpha helix conformation proteins (indicating that the primary binding mode in membranes is hydrophobic), and the second is that the ionic and polar heads of all lipid molecules, as well as the ionic side chains of the protein structure, would be found on the outer surface of the membrane. The protein sequences consisting of nonpolar side chains and hydrocarbon chains of the relatively nonpolar phospholipids would be found in the inside of the membrane. The helical portions of the protein were in the inside of the membrane, where they are stabilized by hydrophobic interactions (Figure 3J) [53].

By 1972 Singer, with experimental thermodynamic bases, added globular proteins to his mosaic model. Furthermore, he mentions that the membrane should be fluid, rather than a crystalline matrix. In addition to this, the membrane would be dynamic and translational diffusion would occur in it in the plane of the membrane (Figure 3L) [54].

##### Micellar Model

Throughout history, some micellar models have been proposed, including Lucy’s model in 1968 [55]. This model considers the thermodynamics of the membranes and solubilization studies, in which it is indicated that there are phospholipid micelles existing within biological membranes that contain inserted globular proteins or lipoprotein molecules inside. It was noted that the proportion of lipid molecules in the micellar configuration could vary, not only from one membrane to another, but also within any membrane, depending on the microenvironment and the chemical constitution of the membrane (Figure 3K).

### 4.3. Dynamics of the Plasma Membrane

#### 4.3.1. Lateral Diffusion of Proteins

**Figure 4:**
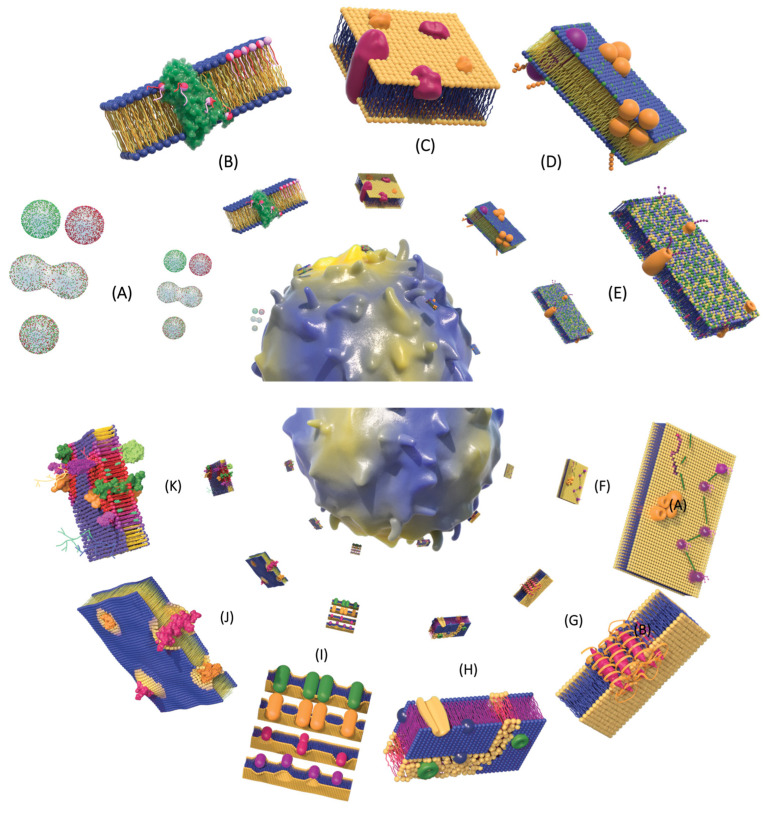
Dynamics of the plasma membrane. (**A**) Frye and Edidin [56] showed in heterokaryons that antigens propagate through the membrane, the PM being “fluid” which allows free “diffusion” of surface antigens. (**B**) Papahadjopoulos and Ohki [57] mentioned that in the membrane there is an inversion of phospholipids, a term that McConnell and Kornberg [58] renamed as flip-flop. (**C**) Singer and Nicolson [59] propose the fluid mosaic model, which contains integral globular membrane and peripheral proteins, which are randomly distributed in the plane of the membrane. (**D**) Gitler [62] in his proposed model of plasticity shows two different types of proteins interspersed in the lipid bilayer: integral and peripheral, and in the proteins, additionally, there were sugar residues of glycoproteins and glycolipids towards the outer membrane. He gives measurements of 8.5 nm in total diameter for proteins and 1 nm in diameter for lipids. (**E**) Bretscher [63] proposes an asymmetric lipid bilayer, with random distribution between the bilayers (represented with different colors): phosphatidylcholine was preferably used in the outer monolayer, and sphingomyelin (which would be enriched with phosphatidylethanolamine and phosphatidylserine) was preferably used in the inner layer. (**F**) The hydrodynamic model of membrane flow [65], shows the lateral diffusion of proteins in membranes (monitoring the protein diffusion with arrows). (**G**) Unwin and Henderson [69,70], by using electron microscopy, observed a protein with a single polypeptide chain with alpha helix conformation and seven alpha helices. (**H**) Jain and White [71] propose that the membrane is separated by ordered and rigid regions of lipids that are separated from each other by fluid and disordered regions (model plate). (**I**) Israelachvili [72] described the behavior of the lipid phase through a simple geometric property of the lipid molecule, which he called the packing parameter. (**J**) Mouritsen and Bloom [73] proposed the Mattress Model: the separation of ordered regionsfrom disordered (fluid) regions occurs in biological membranes as a natural consequence of specific intermolecular interactions and lattice deformation. (**K**) Several authors propose in their models (domains, microdomains, lipid rafts, and membrane rafts) that the rafts are rich in proteins anchored in cholesterol, sphingomyelin, and glycosylphosphatidylinositol (GPI); they have more ordered acyl side chains, and they are thicker than non-raft bilayer domains [74,75,76,77,78].

In the early 1970s, with the evidence of integral proteins of the PM, Frye and Edidin [56] showed that some proteins could easily diffuse into lipid bilayers. To prove this, they used two powerful techniques, one that allowed them to fuse cells from two different species and the other to mark and label cell surfaces. They carried out an experiment where they fused mouse cells with human cells by treatment with the Sendai virus, which produces a hybrid cell called heterokaryon. The mouse cells were labeled with protein-specific antibodies to which they covalently attached a green, fluorescent marker. Proteins from human cells were tagged similarly, but with a red fluorescent marker. At the time of the fusion, the murine and human proteins were observed by fluorescence microscopy to be segregated in the two halves of the heterokaryon. After 40 min at 37 °C, these proteins had completely mixed (Figure 4A). These results provided the first direct evidence that some proteins move laterally in the plane of the PM, a process that slows down with lower temperature.

#### 4.3.2. Flip-Flop

Papahadjopoulos and Ohki [57] worked with an artificial model of membranes constituted of phosphatidylserine under conditions of asymmetric distribution of ions and, therefore, charges on the two faces of the lipid bilayers. They observed that this asymmetry generated an effect on the stability and electrical properties of the membranes. These authors suggested that the instability generated by this asymmetry could be because these conditions generate the inversion of molecules or groups of molecules from one to the other side of the bilayer. The phospholipid inversion proposed by Papahadjopoulos and Ohki was named “flip-flop” by McConnell and Kornberg [58] (Figure 4B), who worked with egg phosphatidylcholine vesicles in an aqueous phase. These vesicles contained a lipid bilayer whose distribution was asymmetric on both sides and changed when sodium ascorbate was added to the medium at 0 °C, since the phosphatidylcholine molecules moved to the other side of the bilayer.

#### 4.3.3. Fluid Mosaic Model

The fluid mosaic model of the structure of cell membranes was formulated by Singer and Nicolson [59]. These authors incorporated simple and well-founded observations, and experimental ideas accumulated between the decades of the 1950s to the 1970s. The model suggested by Singer and Nicolson preserves the basic lipid structure proposed by Gorter and Grendel [18]. The model mentions the viscous bilayer as a sea of lipids, which have hydrophobic surfaces that make them insoluble in an aqueous solution. Surfaces that float with a random distribution on the membrane and may contain channels or pores, to allow the passage of molecules through the membrane. The thermodynamic and experimental bases proposed by Lenard and Singer [53] supported the Singer and Nicolson model, which also posits the existence of globular proteins (integral proteins), as Branton observed in 1966 [49], but with the premise that integral proteins of the membranes have an amphipathic structure in the intact membrane; that is, its ionic and highly polar groups are largely located on the membrane surfaces in contact with the aqueous medium, while its nonpolar residues are sequestered from contact with water inside the membrane. Other proteins that do not cross the membrane, which are called peripheral proteins, are associated with hydrophobic surfaces, which could freely diffuse through the lipid bilayer, as reported in the work of Frye and Edidin in 1970 [56]. The authors also mention in their model that diffusion would not prevent the existence of associations between proteins that could form clusters or specific interactions between themselves or between proteins and a particular lipid species.

On the other hand, the individual lipid molecules could move laterally; endowing this structure with fluidity, as well as high electrical resistance and relative impermeability concerning very polar molecules; indicating that the hydrophobic tails are inward, far from the water. The hydrophilic heads of phospholipids are on the outside where they interact with water molecules in the fluid environment of the cell. For this reason, the authors mention that the mosaic is fluid and dynamic. Its components in the two-dimensional viscous solution can undergo translational and rotational diffusion [59] (Figure 4C). The fluid mosaic model highlights the homogeneous distribution of lipids and proteins, mentioning that the membrane is asymmetric between the hemilayers. After its approach, this model has been subject to constant review and updating [60].

#### 4.3.4. Membrane Plasticity

The concepts published by Singer and Nicolson were shared and published independently, and almost simultaneously, by the Mexican scientist Carlos Gitler [61,62]. Gitler’s model suggests that membranes are a nearly fluid, liquid crystalline lipid bilayer in which the constituent molecules are constrained by the bilayer arrangement. He proposed that there are proteins embedded in or traversing the bilayer, and others interacting primarily in the nonpolar regions of membrane phospholipids. The components of the membrane are not static; they move individually and probably in groups. He called this phenomenon plasticity, in a way that the structures are almost fluid, and the membrane is subject to fluctuations induced by changes in its environment and interaction with other surrounding molecules. Lipids associate with other lipids, as well as between proteins and, in turn, between lipid groups (clusters) of the membrane; the interactions between lipids and proteins would be stabilized by hydrophobic forces, as well as through ion-dipole and dipole-dipole surface forces. Gitler suggested the possibility that in most membranes there is an asymmetric distribution of proteins, and that the main fraction of proteins is present in the inner layer, while in the outer layer there are glycoproteins, glycolipids, and other proteins whose function is not yet defined (Figure 4D). He finally pointed out that the functionality of the membrane elements would depend on the nature of these transitions.

#### 4.3.5. Asymmetric Lipid Bilayer

Another important contribution to the knowledge of the membrane organization was the transverse discovery, proposed in 1972 by Bretscher [63], who provides the first evidence that lipids are not randomly distributed within the bilayer. He determined in studies carried out with ghosts of human erythrocytes, that in the outer monolayer phosphatidylcholine was preferentially found, while in the inner layer sphingomyelin is present, and would be enriched with phosphatidylethanolamine and phosphatidylserine (Figure 4E). With these results, he introduces the concept of membrane lipid asymmetry, which refers to the different composition between the inner (cytoplasmic cis face) and the outer (exoplasmic face) of the lipid monolayer (or leaflets) of the biomembranes, in terms of macromolecular composition [63,64].

#### 4.3.6. Hydrodynamic Model of Membrane Flow

Saffman and Delbrück in 1975 [65] hypothesize that molecular diffusion rates depend mainly on the viscosity and thickness of the membrane, and only weakly on the size of proteins and aggregates. They consider the membrane as an infinite flat thin sheet immersed in an infinite bulk fluid (Figure 4F). Subsequently, corrections to the model were made by Naji et al. [66], who observed that protein–lipid interactions locally deform the membrane, and this deformation generates, in turn, new hydrodynamic stresses in the membrane complex that leads to a suppression of their mobility. In conjunction with the work of Gambin et al. [67], it showed that this suppression is consistently protein size- dependent. A new model, an extension of the Saffman–Delbrück model, described the effects of periodic boundary conditions on the diffusion constants of lipids and proteins obtained by simulation [68].

#### 4.3.7. Secondary Alpha Helix Proteins

Although it was known through previous studies that membranes contained proteins of helical conformation, Unwin and Henderson contributed their experiments (by determining the three-dimensional structure of bacteriorhodopsin, as well as its orientation in the membrane) to confirm that an integral protein consisted of a single peptide chain that folds over and over in the lipid bilayer up to a total of seven times. Each of the transmembrane segments of the protein is a tightly packed alpha helix composed mainly of hydrophobic amino acids. The successive transmembrane segments are anchored to one another by small loops of hydrophilic amino acids that extend and protrude from the polar surfaces of the membrane, confirming the existence of integral proteins (Figure 4G) [69,70].

#### 4.3.8. Plate Model

To account for lipid-mediated lateral heterogeneity, alternative models of biological membranes were proposed. The “plate model”, introduced by Jain and White in 1977 [71], emphasizes that the “membrane continuum is divided into several relative rigid plates or patches that are in motion relative to each other”. According to this model, the ordered and rigid regions are separated from each other by fluid and disordered regions, which occur in biological membranes as a natural consequence of specific intermolecular interactions and lattice deformation. The separation of ordered and relatively disordered regions is lateral, in two dimensions, and can occur in both monolayers of the bilayer (Figure 4H). From the model proposed by Jain and White, an extensive investigation continued on the lipid domains that they had represented as lipid patches.

#### 4.3.9. Geometry of Molecules

Subsequently, some theoretical models were developed that predict the topology of membrane aggregates. The simplest of such models was proposed by Israelachvili in 1977 [72], which stipulated that various lipid–protein interactions could be mutually adjusted by their hydrophobic structures, by the so-called “packing parameter”, which is related to the geometry of the molecule (Figure 4I). According to this model, the association of conical-shaped molecules gives rise to an aggregate with great curvatures, such as micelles. Instead, the association of relatively cylindrical molecules gives rise to aggregates with little curvatures, such as membranes. This model allows us to explain some transitions observed in the phases of amphiphilic molecules. On the other hand, it proposes the existence of lateral interactions between lipids and proteins. Furthermore, he also incorporated into the model membrane folding, pore formation, and variations in its thickness, as well as some degree of lateral heterogeneity.

#### 4.3.10. Mattress Model

The model proposed by Mouritsen and Bloom in 1984 [73], called the “Mattress model”, was designed with mixtures of lipid membranes and amphiphilic proteins or polypeptides in an aqueous solution to describe the behavior of lipid membranes, taking as basic geometric variables the thickness of the region hydrophobicity of the lipid bilayer and the length of the hydrophobic region of proteins. The model suggests that proteins and lipids exhibit interactions associated with positive Gibbs free energy, due to variations in the hydrophobic length of the molecules; whether the length of the hydrophobic core of a membrane protein is longer or shorter than this, the lipid membrane must be deformed or make adaptations to compensate for unfavorable hydrophobic interactions. This effect is called the “hydrophobic effect”, in which the “pairing” of lipids and proteins would give rise to interfacial tensions between them. These tensions would lead to the accumulation of certain lipid species around proteins, and the mutual attraction of proteins due to intramolecular forces, leading to protein aggregation and clumping (Figure 4J) [73].

### 4.4. Membrane Platforms: Domains, Microdomains, Lipid Rafts and Membrane Rafts

Singer and Nicolson described membranes in their model as a uniform, fluid, and relatively homogeneous sea of lipids (iceberg) with a random distribution. Years later, with the study of asymmetric lipid trafficking (assisted mainly by the technique of extraction with nonionic detergents and membrane fractionation), the concept of the existence of multiple phases in the membrane lipid environment was formulated, which can drive the “organization of membrane lipid components into domains”. Karnovsky et al. presented evidence that lipids are organized into domains, whose heterogeneity is functional and structural, formed by lipid–lipid or lipid–protein interactions, with cholesterol being an important component for the formation of membrane phases and domains [74].

In 1988, Simons and van Meer [75] proposed the term lipid rafts microdomains, which they postulated from their studies on the differential distribution of sphingolipids towards the apical membrane of polarized epithelial cells. This model posits that glycosphingolipids are transported asymmetrically to the apical cell membrane, suggesting that sphingolipids and cholesterol are essential for the distribution of protein in the membrane [75,79].

The idea that these rafts, being enriched in cholesterol, should have special physical properties arose from the original observations on model membranes reported by Ipsen et al. [80], who showed that, under particular conditions, cholesterol generates the coexistence of ordered or disordered liquid lamellar phases in a phospholipid bilayer. As the cholesterol interacts differently with the conformational and translational degrees of freedom of phospholipid molecules, a liquid order phase has been proposed [80].

The concept of lipid microdomains was modified by Simons and Ikonen in 1997 [76], in which they proposed the term “lipid rafts” as high molecular order PM platforms enriched with cholesterol, glycosylphosphatidylinositol (GPI), and sphingolipids on the exoplasmic face of the bilayer, creating floating phospholipid platforms. Hence the name, in which proteins involved in signal transduction can selectively interact with effector molecules (Figure 4K) [76].

The concept of “lipid rafts” was replaced by the understanding that proteins and lipids contribute to these membrane microdomains. At the Keystone Symposium on Lipid Rafts and Cellular Function, researchers from different areas of biology agreed on the definition of membrane rafts: membrane rafts are small (10–200 nm), heterogeneous, highly dynamic, sterol- and sphingolipid-enriched domains that compartmentalize cellular processes. Small rafts can sometimes be stabilized to form larger platforms through protein–protein and protein–lipid interactions [77].

### 4.5. Membrane Interaction: Glycocalyx–Membrane–Cytoskeleton

#### 4.5.1. Membrane: A Three-Layer System Composed of Glycocalyx–Membrane–Cytoskeleton

**Figure 5:**
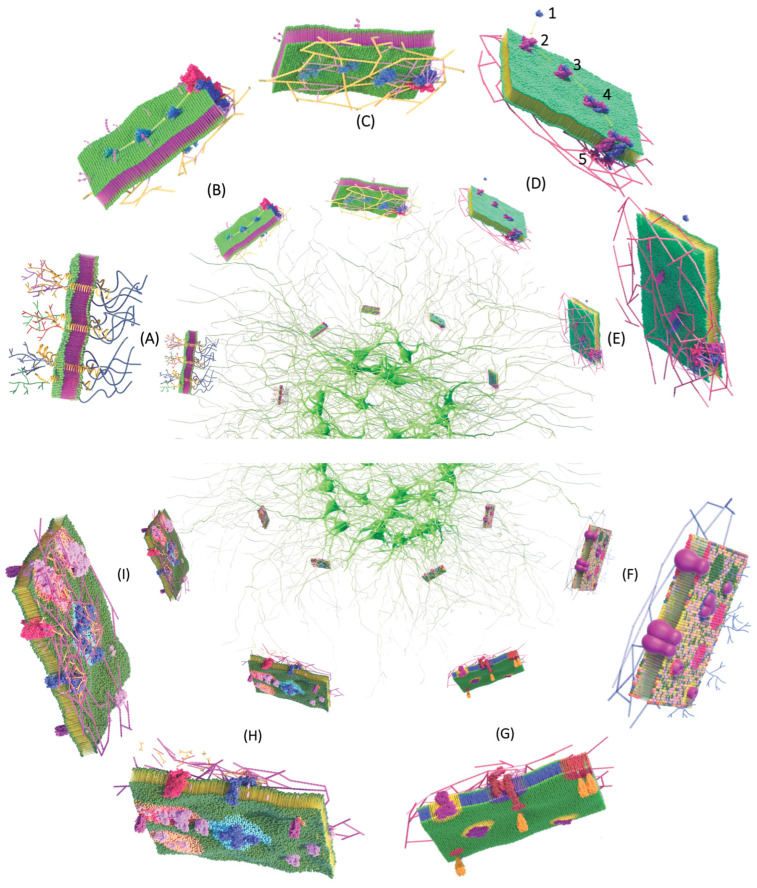
Membrane-cytoskeleton interaction. (**A**) The model proposed by Sackmann [81] mentions that the plasmatic membrane is a system composed of three layers, whose center is formed by a liquid-crystalline bilayer of lipids and proteins. On the outside it is covered by the glycocalyx: a macromolecular film formed by oligosaccharides associated with lipids and oligosaccharides branched from proteins called glycoproteins. (**B**,**C**) Kusumi et al. [83,86] propose the Picket-fence model: confinement zones are formed through non-specific diffusion barriers of transmembrane proteins with their immediate lipid environment (pickets) and filaments of the cytoskeleton that lie close and parallel to the plasma membrane (fence). Molecules undergo “hop diffusion” when they cross the fence into a neighboring area. (**D**,**E**) Kusumi and Sako [95], Iino et al. [98], and Kusumi et al. [86] propose the oligomerization-induced entrapment model: a ligand (1) binds to an integral membrane receptor protein (2). The receptor with the ligand diffuses until it meets a similar molecule to form a dimer (3), which slows its rate of transition between compartments (4). Signaling molecules, both membrane-bound and cytoplasmic, begin to accumulate around the receptor molecule forming a signaling complex (5). The signal complex is now locked in a compartment due to its size (i.e., it can no longer escape through the fence and/or posts). (**F**) The biomembrane represents for Escribá et al. [100] an asymmetric membrane of lipids and microdomains enriched in particular lipids and those induced by membrane proteins, in addition to the associated cytoskeleton. (**G**) Lillemeier et al. [101,102] and Lillemeier & Davis [103] propose that all plasma membrane proteins (100%) segregate into different membrane domains, called protein islands, depending on their function and nature. The domains are enriched with cholesterol and associated with the actin cytoskeleton, which regulates their positioning, separation, and/or fusion. Islands can be subdivided into raft and non-raft islands, which is also illustrated by their lipid composition (bright green and dark green lipids, respectively) and protein content (brown and yellow proteins, respectively) (**H**,**I**). Gowrishankar et al. [104] proposed the model of active actin, in which F-actin esters formed just below the plasma membrane drive lipid-anchored proteins into nanoclusters, perhaps through myosin-based contraction.

The model proposed by Sackmann [81] mentions that the plasma membrane is a system composed of three layers, whose center is formed by a liquid-crystalline bilayer of lipids and proteins. On the outside it is covered by the glycocalyx: a macromolecular film formed by oligosaccharides associated with lipids and oligosaccharides branched from proteins called glycoproteins (Figure 5A). The glycocalyx can protrude several tens of nanometers into the extracellular space, as previously described [41,42]. On the intracellular side, the bilayer is coupled to the membrane-associated cytoskeleton, as a nearly two-dimensional macromolecular network. In most cells, the inner lamina of the membrane is coupled to the three-dimensional macromolecular network of actin filaments [81] or associated with talin [82].

#### 4.5.2. Membrane–Cytoskeleton Interaction, Picket-Fence Model

Several authors have studied the movements of lipids and proteins in the lateral plane of the PM and their interaction with the actin cytoskeleton. The union of two PM models generated the picket-fence model, which is named for being the union of two models called the “membrane skeleton fence model” and the “anchored-protein picket model” [83,84,85,86]. This model explains the suppressed diffusion of proteins and lipids in the PM, suggesting that there are “confinement zones” in the PM (ranging from 30 to 250 nm in diameter) where various transmembrane proteins are anchored and aligned along the PM backbone. These transmembrane proteins effectively act as posts/stakes (pickets) in rows that prevent the diffusion of phospholipids. The transmembrane proteins protrude into the cytoplasm, and their cytoplasmic domains collide with the actin backbone which induces temporary entrapment or fencing of the transmembrane proteins. Proteins can jump between adjacent compartments when the distance between the network or the membrane is long enough or when the network dissociates temporarily or locally (Figure 5B,C) [87,88].

#### 4.5.3. Oligomerization-Induced Entrapment Model

The redistribution and clustering of receptors are key in many signal transduction pathways [89,90,91]. Several reports indicate the active role that the cytoskeleton plays in inhibiting or enabling the redistribution or assembly of membrane molecules [92,93,94]. Based on the above, the oligomerization-induced trapping model explains that monomers temporarily confined in the PM can jump from one compartment to another with relative ease. When a receptor molecule receives an extracellular signal, receptor oligomerization is induced, leading to the recruitment of downstream signaling molecules to the cytoplasmic tail of receptors; however, after oligomerization, the newly formed oligomer must jump over the compartment boundaries (fences) as a whole rather than the monomers. For this reason, the oligomer will require a larger and longer-lasting gap to cross a compartment boundary. This would result in a longer residence time in either compartment since it would correspond to a decrease in the long-range diffusion rate of the oligomer. Due to the trapping effect induced by oligomerization, the membrane skeleton would temporarily trap the newly formed complex within the same compartment where the extracellular signal was received (Iino, 2001). The effect of temporary confinement of molecules on oligomerization (or complex formation) has been called “oligomerization-induced entrapment” (Figure 5D,E) [95,96,97]. Such temporary immobilization due to oligomerization has been observed for the immunoglobulin E receptor FcεRI and the E-cadherin [98,99].

#### 4.5.4. The Vision of Escribá et al., (2008)

One of the contributions to the concept of the membrane was the vision of Escribá et al. [100]. The authors mention that there is a great diversity of lipids forming specialized domains around the integral proteins of the membrane and the glycoproteins, which in turn are asymmetrically distributed in the membrane (Figure 5F), as mentioned by Bretscher [63].

#### 4.5.5. Protein Island Model

This model, based on super-resolution electron microscopy studies, proposes that PM proteins (100%) are segregated within different domains called rafts (protein islands), according to their function and nature. These domains are enriched for cholesterol and associated with actin filaments of the cytoskeleton, which regulates the position, separation, and/or coalescence. This model was made based on T-cell observations where lipid rafts are described as a subpopulation of lipid islands, and protein islands (PI) in nanoclusters in the membrane, that can be differentiated into non-raft and raft, based on the characteristics of the molecules associated with it.

Protein-free and low-cholesterol regions of the membrane can separate PI. In this case, “hop diffusion” is postulated to be the translocation of a molecule from one PI to another across shared temporal boundaries. Therefore, transient confinement is due to protein localization in PI (Figure 5G) [101,102,103].

#### 4.5.6. Active Membrane–Actin Composite Model

Another contribution to the knowledge of the membrane is “the active actin–membrane composite model” proposed by Gowrishankar et al. in 2012 [104]. This is a theoretical and experimental model, which shows evidence from TIRF and FCS microscopy (Total Internal Reflection Fluorescence and Fluorescence Correlation Spectroscopy). In this model, GPI-anchored proteins in the exoplasmic portion are organized into immobile and monomeric nanoclusters. This lipid microenvironment, also formed by clusters of proteins and glycolipids, is important for signal transduction. The nanoclusters are dynamic, with high rates of aggregation and defragmentation, dependent on actomyosin activity. The nanoclusters fragment into monomers, in which the cell membrane detaches from the cortical actin cytoskeleton. Therefore, the molecular organization and dynamics are driven by the cortical actin network, formed by cross-linked actin filaments, stable, and tangentially oriented to the PM, punctuated by actin bundles. Cortical actin forms a meshwork that influences the spatial pattern of membrane components. In addition to this stable meshwork, there is a population of short, dynamic actin filaments subject to actomyosin contractility (and thus ATP-dependent) (Figure 5H,I).

In 2015, Raghupathy and colleagues [105] demonstrated that GPI-anchored proteins associate transbilayer in the following manner: the acyl chain portion of the GPI in the outer bilayer couples with cholesterol, and the long acyl chains of the lipids in the inner bilayer. They identified phosphatidyl serine as the inner membrane lipid that allows coupling, and cholesterol as a key element to keep the transbilayer association stable.

#### 4.5.7. Griffié, Peters y Owen (2020)

Because protein nanoclusters have been mentioned to be important for cell signaling, several research groups have been given the task of designing computer models for the study of nanoclusters with the agent-based modelling technique [106]. The authors analyzed the clustering of PM molecules. They hypothesize that the clusters depend on a single parameter that applies to each agent, called the “clustering desire” of the molecules, which is the result of a combination of competing for cellular processes ranging from self-affinity, membrane lipid domain, size, and the Picket-fence model. They mention that the clustering depends on the density of the agent’s molecular environment and its diffusion coefficient. As a result of their tests, they found that the clustered distributions obtained through simulation resemble clusters found in cells and that faster-diffusing molecules result in a clustered distribution that differs from slow-diffusing molecules. They consider the actin meshwork directly below the PM throughout the time course and find that actin polymerization and organization in regions of the dense meshwork are associated with more numerous but, more importantly, less dense clusters. These processes have already been observed in cells, so they consider that agent-based modelling can predict the macroscale results of PM perturbations and can be used to study membranes.

## 5. Conclusions

Throughout this review, we have seen how the concept of the membrane has evolved, mainly due to two facts: firstly, the advancement of biochemical and biophysical techniques, and secondly, the conceptual changes and ideas of researchers who have allowed a more adequate interpretation of membrane concepts and a reinterpretation of old concepts. When by good fortune these two kinds of events have come together, progress has been particularly rapid.

The study of the membrane can be divided historically before and after the model of Singer and Nicolson [59]. The first studies were based on the analysis of the properties of the cell surface, in which they tried to determine the passive profile of permeability, in that certain types of molecules could diffuse freely into a cell, and other types could not. After this, studies were carried out on the composition and architecture of the membranes, determining that they consisted of a lipid bilayer. These studies were simply extraordinary, considering the bilayer as the basis of the studies by Gorter and Grendel [18]. Once it was determined that there were embedded proteins in the bilayer, they were integrated into the lipid bilayer model [49] and were no longer considered merely superficial, as previously proposed by Danielli and Davson in 1935 [27].

During the years 1935–1972, together with the characterization of the cell membrane as a lipid bilayer with proteins embedded in it, the following terms were conceptualized: dynamics, fluidity, elasticity and asymmetry of the membranes, the existence of different types of lipids in the endoplasmic and exoplasmic faces, as well as the different structure of proteins. Another element visualized by electron microscopy was also described: the glycocalyx.

After the structure of the membranes became known, studies of the lateral movement of proteins through the plane of the membrane began, with the experiment of Frye and Edidin [56]. Later, Gitler’s models were published, as well as the Singer–Nicolson model [59].

Subsequent to these models, and with other study techniques, the existence of domains and lipid rafts were proposed, these sites being necessary for signal transduction. On the other hand, investigation of protein–lipid interactions was carried out, finding that lipids bind with a certain affinity and specificity to certain proteins (due to the presence of lipid-binding domains in them). Recent membrane studies have focused on protein–protein, protein–lipid interactions, and interaction with the cytoskeleton and extracellular matrix, as well as lateral movement of lipids and proteins using powerful single-molecule tracking techniques.

In conclusion, the composition and supramolecular architecture of PM is directly related to the multiple functions of biological membranes, in addition to serving as a cell boundary and matrix or scaffold. Biological membranes serve as organizers of multimolecular interactions that are dynamic in time and physical space, and functionally active.

There is a great diversity of reviews on the history and models of PM. In this review, not all existing models were considered; only those that provided the most important contributions, the most controversial, or those that were thought correct due to recognition of their authors were considered. In case of having a special interest in other PM models suggested in the past, we can recommend the review carried out by Stoeckenius and Engelman [107], Jain and White [71], and de la Serna et al. [108]. Models based solely on computational simulation were not considered either.

## 6. Final Considerations

**Figure 6:**
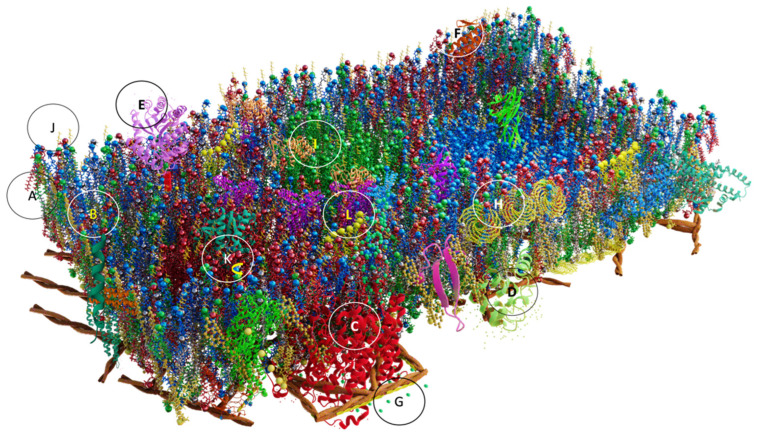
The plasma membrane. (**A**) Asymmetrical distribution of lipids in the bilayer: the outer leaflets contain neutral phospholipids, such as sphingomyelin and phosphatidylcholine, and the inner leaflet contains anionic phospholipids, such as phosphatidic acid, phosphatidylserine, phosphatidylethanolamine, and phosphatidylinositol species (such as phosphatidylinositol 4,5-bisphosphate and phosphatidylinositol triphosphate). The asymmetry of membrane proteins is outlined below: (**B**) a single alpha helix, (**C**) multiple alpha helices (bacteriorhodopsin), (**D**) anchored to the cytosolic surface of the lipid bilayer, proteins that are attached to the bilayer only through single-strand covalent attachment either to a fatty acid or a prenyl group of the cytosolic monolayer, (**E**) through an oligosaccharide bridge, or to a phosphatidylinositol of the non-cytosolic glycosylphosphatidylinositol (GPI) monolayer, and/or (**F**) proteins associated with other membrane-embedded proteins linked through extracellular or intracellular non-covalent interactions. (**G**) Asymmetry is also achieved by intracellular and extracellular ion concentration mediated by proteins such as aquaporin. (**H**) The curvature of the membrane is given by lipids and proteins with an intrinsic or inverted conical shape. (**I**) Lipid and protein islands. On the outside, (**J**) the glycocalyx. (**K**) Rotational and (**L**) transverse lipid and protein movements.

**Figure 7:**
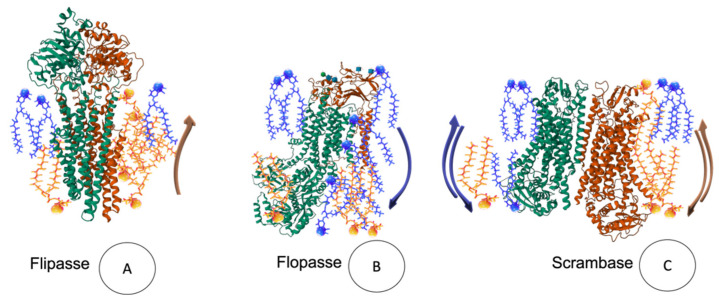
Flip-flop movement mediated by flippases (**A**), which transport lipids from the outer to the cytosolic leaflet, floppases (**B**) that move lipids in the opposite direction, and scramblases (**C**) that mediate bidirectional transport.

**Figure 8:**
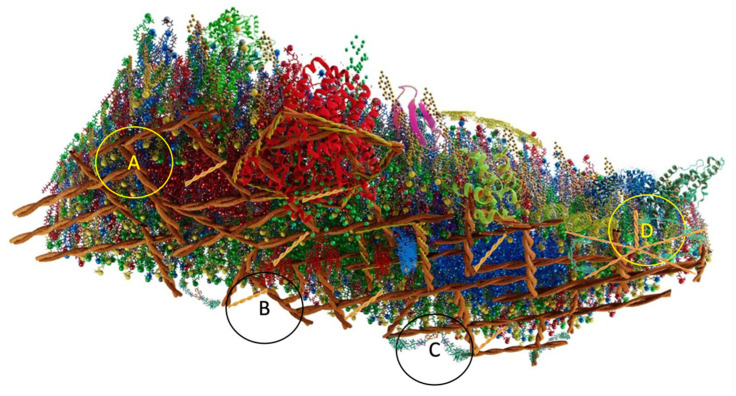
The lipid bilayer is associated on the cytosolic side by means of actin (**A**), active actin (**B**) and myosin (**C**). Picket-fence aster (**D**). Asters are composed of short dynamic F-actin filaments arranged in the form of spokes around a nucleation center containing the complex.

As seen throughout this review, various important concepts have been added about the structure-function of PM. To date, new discoveries continue to appear that add to the lipid bilayer model, made with both experimental and computational data, contributing to the knowledge of the membrane. Since cellular organization is essential for proper cell function, it is important to jointly understand its constituent components and associated structures to appreciate the functions of the PM. As a summary, we take up the concepts that we believe are relevant and generalize them in several schemes considering the evolution of the PM concept (Figure 6, Figure 7 and Figure 8).

Biological membranes in aqueous media form spontaneously adopting the lipid bilayer form (Figure 6A), since thermodynamically the energy provided by the hydrophobic effect and van der Waals forces allows them to form bilayers, made up of various species of lipids that are distributed asymmetrically (Figure 6B) [109]. Many lipids are not distributed homogeneously as they enrich specific compartments or even subregions of a compartment in the PM at a particular site forming raft lipids (Figure 6I) [110]. On the other hand, phospholipid unsaturation is dramatically asymmetric, with the cytoplasmic sheet being approximately twice as unsaturated as the exoplasmic sheet [111] (Figure 6A).

Associated with the lipid matrix are proteins in a proportion of 25 to 75% of the mass of the PM [112]; this variability occurs between the various cell types. The structural asymmetry of the PM is reflected in the asymmetric structures of the transmembrane domains of proteins and/or localization: the integral proteins interact with lipids where they can establish hydrophobic and hydrophilic interactions with their respective lipid counterparts. Peripheral proteins are associated with the membrane mainly by electrostatic interactions with groups of lipid heads (Figure 6F). These interactions are transitory, given their function as signaling, stability, and curvature complexes, among others. Various proteins are bound to proteins or lipids and are not associated with the hydrophobic matrix (Figure 6D,E) [113].

Other components of PM are glycoproteins and glycolipids, proteins or lipids that are covalently attached to carbohydrate chains in the form of oligosaccharides located in the extracellular leaflet called glycocalyx, generating asymmetry. The glycocalyx extends 150 to 400 nm outside the membrane and is arranged in 100 nm wide units along the plane of the membrane (Figure 6J). The main function of the glycocalyx is to act as a barrier between the cell and its environment, but it is also involved in the mechano-sensing [114,115,116].

Lipids and proteins generate a structural asymmetry; however, it is considered that ions also generate an asymmetry because one of the functions of PM is the transport of ions and molecules that pass through proteins, for example, aquaporins (Figure 6C,G). The concentration of these ions generates a gradient that drives a series of cellular processes that impacts the electrostatic interactions of charged proteins [117].

The movement of lipids and proteins generates plasticity and/or flexibility that causes the membrane to be curved. The membranes given by certain lipids with a non-conical shape, for example, can deform the flat structure of lipid bilayers when they bend and thus modify their curvature. On the other hand, transmembrane proteins (ion channels, transporters, and receptors) that have an intrinsic conical or inverted conical shape can fold their associated membranes (Figure 6H) [118,119,120].

The components of the PM are not static structures, they are otherwise dynamic, and fluid given by lipids, proteins and fatty acids, and events mediated by three modes of movement: rotational, translational, and transbilayer [121] (Figure 6K,L and Figure 7). The rotation movement of proteins and lipids occurs essentially around the axis perpendicular to the plane of the membrane, and lateral diffusion occurs along the plane of the membrane [56]; this movement also includes the anchoring of actin, protein of the cytoskeleton. Finally, transbilayer diffusion or flip-flop is the movement of only phospholipids that occurs between the halves of the bilayer, being a slow and unfavorable process from the energetic point of view [58,121]. However, lipid flip-flop rates can be greatly accelerated by lipid transporters called flippases, flopasses, and scramblases (Figure 7A–C) [122,123].

The interactions of the PM with the cytoskeleton are mediated by cortical proteins that are driven by processes that consume ATP, in which active actin participates (Figure 8A), and where actin filaments polymerize and depolymerize continuously (Figure 8B) [124,125]; in addition to being persistently acted upon by a variety of myosin motors (Figure 8C) [126,127]; and exert contractile stresses on cortical actin filaments, continuously remodeling the architecture of the cortex of the PM. Polar actin filaments and myosin-dependent contractility and alignment drive these filaments in spontaneous patterns that include inward-pointing “asters” (Figure 8D) [128,129]. These active processes, in turn, can generate tangential stresses and currents at the cell surface, which could drive the dynamics and local composition of membrane components at different scales [130].

Because of its biological importance, the membrane continues to be studied using increasingly sophisticated experimental techniques. Experimental advances include improved methods such as single particle tracking, fluorescence correlation spectroscopy, super-resolution imaging, scattering, solid-state nuclear magnetic resonance, and mass spectrometry, as well as methods for asymmetric model membranes and extracts of real cell membranes. In addition, through computer simulations that have provided some details, and through molecular dynamics techniques, studies are able to describe the interactions between all the components of the system at the atomic resolution level, acting as a “computing microscope” [131].

Finally, and as mentioned by de la Serna et al. [108]: “There is no simple model of the plasma membrane organization” given by the interactions of its components and between them, as well as the interaction with other intra- and extracellular molecules. The contributions to the concept of the membrane, still not being accurate, were reinterpreted and added to the knowledge of the complexity of PM, seeking to add the knowledge that allows understanding of the functions of the PM.

## Data Availability

The data presented in this study are available on request from the corresponding author.
